# Effects of structured step-aerobics interventions on psychological well-being in nonclinical populations: a systematic review

**DOI:** 10.3389/fpsyg.2026.1843115

**Published:** 2026-06-03

**Authors:** Xiaoxue Gao, Chengcang Li, Renqing Silang, WeiJia Zhou, Yu Shu

**Affiliations:** 1 Pukyong National University, Busan, Republic of Korea; 2 School of Physical Education and Health, Sichuan Technology and Business University, Meishan, China; 3 Aba Teachers College, Aba Tibetan and Qiang Autonomous Prefecture, Aba, China; 4 Chengdu Shuangliu Experimental Primary School East District, Chengdu, China

**Keywords:** exercise enjoyment, exercise psychology, quality of life, sleep quality, step aerobics

## Abstract

**Objective:**

To identify, appraise, and narratively synthesize the effects of structured step-aerobics interventions on psychological well-being in nonclinical populations.

**Methods:**

The eligibility criteria included intervention studies in nonclinical human samples evaluating structured step aerobics and reporting quantitative psychological or psychosocial outcomes. PubMed, Web of Science Core Collection, and Scopus were searched. The RoB 2 or ROBINS-I were used to assess the risk of bias. Results were synthesized narratively.

**Results:**

Seventeen studies were included (10 randomized, 7 nonrandomized; 11 repeated programs, 6 acute studies). Across this small and evidence base, preliminary favorable signals were observed for sleep quality, quality-of-life or general mental-health outcomes, depressive symptoms in one menopause-focused study, and some acute anxiety-related responses, but these findings remain uncertain because all included studies were at high or serious risk of bias. Findings for body image, self-perceptions, enjoyment, and socially evaluative outcomes were mixed and context dependent. All randomized studies were at high risk of bias and all nonrandomized studies at serious risk of bias.

**Conclusion:**

Current evidence is insufficient to determine whether structured step aerobics improves psychological well-being in nonclinical populations. The available studies suggest areas for further investigation, particularly sleep quality, broader perceived well-being, and acute psychological responses, but adequately powered and better-reported trials are needed before effectiveness or practical recommendations can be established.

**Systematic review registration:**

https://osf.io/5kgvd, registered in Open Science Framework (osf.io/5kgvd; 17/03/2026).

## 1 Introduction

Physical activity and exercise are related but distinct constructs, with exercise defined as planned, structured, and repetitive bodily movement intended to improve or maintain physical fitness (Caspersen et al., 1985). A broad literature now indicates that exercise contributes not only to physical health but also to better mood states, health-related quality of life, and other indicators of well-being (Penedo and Dahn, 2005; Mahindru et al., 2023). Step aerobics is a structured class-based form of aerobic exercise in which participants perform choreographed stepping sequences on a raised platform, usually under instructor guidance and at a prescribed intensity (Kennedy and Newton, 1997; Hale and Raglin, 2002). Because this modality combines aerobic load, coordination, external pacing, music, and group participation, its psychological effects may not be identical to those of less structured or more solitary exercise formats (Kerschan-Schindl et al., 2002; Gwyther et al., 2018).

High-level evidence suggests that physical activity reduces depression and anxiety in nonclinical adult populations and is effective for improving depression, anxiety, and distress across diverse adult groups (Rebar et al., 2015; Singh et al., 2023). In nonclinical populations, the relevance of structured exercise is not limited to symptom reduction since it also concerns health promotion, prevention, and the maintenance of psychological functioning through domains such as mood, subclinical distress, sleep, self-perception, enjoyment, and perceived quality of life (Rebar et al., 2015) This health-promotion perspective differs from clinical or rehabilitation contexts, where psychological outcomes may be strongly influenced by diagnosis-specific pathophysiology, symptom severity, comorbidity, medication use, functional limitations, contraindications, therapeutic goals, and concurrent care (Thompson et al., 2020). Accordingly, evidence derived from nonclinical samples offers a more coherent basis for examining whether structured step aerobics is psychologically relevant as a community-based exercise modality rather than as a disease-specific treatment. Group-based exercise contexts may additionally support adherence and psychosocial benefit through enjoyment, social interaction, and a stronger sense of connectedness (Beauchamp et al., 2018; Gwyther et al., 2018). These considerations are relevant because psychological responses to exercise extend beyond clinical symptom reduction to include affective responses, perceived stress, sleep, self-perceptions, and mental components of quality of life (Penedo and Dahn, 2005; Mahindru et al., 2023), which generally can be classified as positive in psychological well-being.

In this context, psychological well-being can be used as an organizing analytical framework rather than as a claim that all included outcomes represent the same construct or the same temporal level of response (Fox, 1999; Penedo and Dahn, 2005; Kredlow et al., 2015). Acute mood, affect, state anxiety, and perceived stress can be considered proximal psychological responses because they may change during or immediately after an exercise exposure, whereas sleep quality was considered a recovery-related outcome that may reflect downstream changes in arousal, fatigue, and restoration after repeated exercise exposure (Fox, 1999; Penedo and Dahn, 2005; Kredlow et al., 2015). Self-perceptions, enjoyment, intention to continue participation, and mental-health-related quality-of-life domains can be considered as broader psychosocial or integrative well-being outcomes because exercise research commonly evaluates well-being across affective, self-evaluative, behavioral, and perceived-functioning domains (Penedo and Dahn, 2005; Marquez et al., 2020).

Within step-aerobics-specific studies, transient mood states improved after bench-stepping classes in healthy adults, and acute state anxiety reductions have been reported across repeated weeks of participation (Kennedy and Newton, 1997; Hale and Raglin, 2002). Step-aerobic sessions have also been described as pleasurable in untrained adults, and enjoyment appears to be enhanced when the class climate and instructor behavior are socially supportive (Fox et al., 2000; Kerschan-Schindl et al., 2002). Over longer intervention periods, step aerobics has been associated with improved sleep quality in adolescents and with improved general health perception or quality-of-life domains in community-dwelling older adults (Dunsky et al., 2017; Hu et al., 2023). The available literature therefore suggests that step aerobics may influence both immediate affective responses and broader well-being outcomes, but the evidence base remains scattered across age groups, settings, and outcome constructs (Kennedy and Newton, 1997; Hale and Raglin, 2002; Hu et al., 2023). Thus, the available step-aerobics literature spans at least three distinct but exercise-relevant outcome levels, namely, immediate psychological responses, recovery-related outcomes such as sleep quality, and broader psychosocial or quality-of-life indicators. These domains are relevant since they collectively describe the psychological and psychosocial relevance of structured step aerobics in nonclinical populations, while being relevant to being interpreted separately according to outcome domain and intervention time horizon.

Despite this emerging evidence, adjacent reviews have focused on physical activity and mental health in general, dance movement therapy and dance interventions, aerobic dance and cognition in older adults with mild cognitive impairment, or dance-related body image and social physique anxiety rather than step aerobics as a distinct exercise modality (Koch et al., 2019; Zhu et al., 2020; Singh et al., 2023; Liu et al., 2025). Reviews of dance interventions in older adults have also aggregated heterogeneous movement modalities and emphasized physical-health outcomes, making it difficult to isolate the psychological relevance of structured step-aerobics programs (Hwang and Braun, 2015; Koch et al., 2019). Consequently, the literature still lacks a modality-specific synthesis clarifying which psychological well-being outcomes have been studied in structured step aerobics, in which nonclinical populations, and under what intervention characteristics.

The objective of this systematic review is to provide a modality-specific synthesis identifying, critically appraising, and narratively summarizing the evidence on structured step-aerobics interventions and psychological well-being in nonclinical populations. A secondary objective is to examine whether intervention characteristics, including exposure duration, program length, class context, and comparator type, appear to influence the direction, magnitude, and consistency of psychological outcomes. The review also aims to map where evidence is concentrated across outcome domains, population groups, and intervention time horizons, and where important gaps remain. The novelty of the present synthesis is therefore its modality-specific focus on structured step aerobics as a distinct exercise format, rather than on aerobic exercise, dance, or group exercise more broadly. It also specifically maps psychological and psychosocial outcomes in nonclinical populations according to outcome level, population group, intervention time horizon, and delivery characteristics. This approach is needed because step aerobics combines aerobic load, choreographed movement, music, instructor cueing, external pacing, and group context, and these features may shape psychological responses differently from less structured aerobic exercise or broader dance-based interventions.

## 2 Methods

### 2.1 Review design and protocol registration

This systematic review was conducted and reported in accordance with the PRISMA 2020 (Page et al., 2021) statement and the associated explanation and elaboration guidance, with literature-search reporting aligned to PRISMA-S (Rethlefsen et al., 2021) and narrative synthesis structured according to Synthesis without meta-analysis (Campbell et al., 2020). The review addressed the question: what are the effects of structured step-aerobics interventions on psychological well-being in nonclinical populations?

The review protocol was finalized *a priori* and registered in Open Science Framework (osf.io/5kgvd; 17/03/2026) before formal study selection begins. No amendments were made after registration.

### 2.2 Eligibility criteria

#### 2.2.1 Population

Studies were eligible if they enrolled human participants from nonclinical populations, including healthy children or adolescents, university students, working-age adults, and community-dwelling older adults. Studies conducted exclusively in samples recruited because of a diagnosed psychiatric disorder, neurological disorder, cardiovascular or metabolic disease, cancer, chronic pain, pregnancy-related condition, formal rehabilitation need, or elite athletic status were excluded because these populations represent clinical, disease-management, rehabilitation, or performance-specific contexts in which psychological outcomes may be influenced by diagnosis, symptom severity, medication use, functional limitations, contraindications, concurrent care, or rehabilitation-specific goals.

#### 2.2.2 Intervention

Eligible interventions were structured step-aerobics as the primary exercise modality. Both acute single-session exposures and repeated programs were eligible, provided that the protocol clearly involved a raised step or bench and a planned stepping routine delivered in a class-based, supervised, or explicitly prescribed home-based format. Studies in which step activity was used only as a fitness test, an outcome assessment, a pedometer target, a stair-climbing intervention, or an exergaming task were excluded.

#### 2.2.3 Comparator

Any comparator was accepted, including inactive control, usual activity, wait-list, attention control, or another exercise modality. Single-group pre-post studies were also eligible because the step-aerobics psychology literature was expected to be small and methodologically heterogeneous.

#### 2.2.4 Outcomes

Studies had to report at least one quantitative psychological or psychosocial outcome measured immediately after an acute session, after a multi-session intervention, or at follow-up. Primary outcomes included mood or affect, anxiety, stress, depressive symptoms, and global psychological well-being. Secondary outcomes included sleep quality, mental-health-related or psychosocial domains of quality of life, enjoyment, self-perceptions, intention to continue participation, and other closely related psychosocial responses. For synthesis, these outcomes were not treated as interchangeable indicators of a single latent construct. Instead, they were grouped by analytical level and time horizon as acute psychological responses, recovery-related outcomes, or broader psychosocial and quality-of-life outcomes.

#### 2.2.5 Study designs

Randomized controlled trials, nonrandomized controlled trials, quasi-experimental studies, controlled before-after studies, and single-group pre-post intervention studies were eligible. Cross-sectional studies, case reports, qualitative-only studies, conference abstracts without full reports, protocols, editorials, letters, narrative reviews, and systematic reviews were excluded.

### 2.3 Information sources

Electronic searches were conducted in PubMed, Web of Science Core Collection, and Scopus on 23 March 2026, from database inception to the search date. To improve retrieval completeness without substantially broadening the review beyond peer-reviewed intervention evidence, the reference lists of all included studies and of closely related review articles were screened manually for additional eligible reports. Forward citation tracking was undertaken within Web of Science and Scopus for all included studies when feasible. No language or date limits were applied at the search stage. Non-English full texts identified as potentially eligible were translated sufficiently for screening and data extraction whenever feasible.

### 2.4 Search strategy

The search strategies were developed from preliminary PubMed scoping of step-aerobics studies and adjacent review literature, then translated across databases while preserving the same core concepts: step-aerobics modality terms, and psychological well-being outcome terms. The exact search strategy for each database is presented in Table 1.

**Table 1:** Search strategy in each database.

Database	Search strategy
PubMed	(“step aerobics”[Title/Abstract] OR “step aerobic”[Title/Abstract] OR “step aerobic exercise”[Title/Abstract] OR “aerobic step”[Title/Abstract] OR “bench-step exercise”[Title/Abstract] OR “bench-step exercise”[Title/Abstract] OR “bench-step aerobics”[Title/Abstract] OR “bench-step aerobics”[Title/Abstract] OR “bench stepping”[Title/Abstract] OR “step exercise”[Title/Abstract] OR “stepping exercise”[Title/Abstract] OR “step training”[Title/Abstract]) AND (“psycholog*”[Title/Abstract] OR “psychological well-being”[Title/Abstract] OR “wellbeing”[Title/Abstract] OR “well-being”[Title/Abstract] OR “affect*”[Title/Abstract] OR “mood”[Title/Abstract] OR “anxiety”[Title/Abstract] OR “stress”[Title/Abstract] OR “depress*”[Title/Abstract] OR “quality of life”[Title/Abstract] OR “health-related quality of life”[Title/Abstract] OR “general health perception”[Title/Abstract] OR “sleep”[Title/Abstract] OR “sleep quality”[Title/Abstract] OR “enjoyment”[Title/Abstract] OR “self-efficacy”[Title/Abstract] OR “self-esteem”[Title/Abstract] OR “adherence”[Title/Abstract] OR “intention*”[Title/Abstract])
Web of Science	[“step aerobics” OR “step aerobic” OR “step aerobic exercise” OR “aerobic step” OR “bench step exercise” OR “bench-step exercise” OR “bench step aerobics” OR “bench-step aerobics” OR “bench stepping” OR “step exercise” OR “stepping exercise” OR “step training” OR “step class” (Topic) and psycholog* OR “psychological well-being” OR wellbeing OR “well-being” OR affect* OR mood OR anxiety OR stress OR depress* OR “quality of life” OR “health-related quality of life” OR “general health perception” OR sleep OR “sleep quality” OR enjoyment OR “self-efficacy” OR “self-esteem” OR adherence OR intention* (Topic)] Scopus
Scopus	(TITLE-ABS-KEY (“step aerobics” OR “step aerobic” OR “step aerobic exercise” OR “aerobic step” OR “bench step exercise” OR “bench-step exercise” OR “bench step aerobics” OR “bench-step aerobics” OR “bench stepping” OR “step exercise” OR “stepping exercise” OR “step training” OR “step class”) AND TITLE-ABS-KEY (psycholog* OR “psychological well-being” OR wellbeing OR “well-being” OR affect* OR mood OR anxiety OR stress OR depress* OR “quality of life” OR “health-related quality of life” OR “general health perception” OR sleep OR “sleep quality” OR enjoyment OR “self-efficacy” OR “self-esteem” OR adherence OR intention*))

Because no dedicated indexing term adequately captures step aerobics as a distinct exercise modality across all three databases, the final strategies rely primarily on text words in titles, abstracts, and keywords. Search strings were rerun exactly as documented below on 23 March 2026 and exported in full for reproducibility.

### 2.5 Selection process

All records were exported to EndNote for reference management and deduplication, after which the de-duplicated library was imported into Rayyan for title/abstract and full-text screening.

Two authors independently screened records in two stages. First by title and abstract, and then by full text. Before formal screening began, both authors piloted the eligibility criteria on a calibration sample of records and refined operational decision rules where necessary without changing the substantive criteria.

Disagreements at either stage were resolved by discussion. If consensus was not reached, a third author arbitrated. Reasons for exclusion at the full-text stage were recorded. No machine-learning or other automation tools were used to replace human eligibility decisions.

### 2.6 Data collection process

A standardized data extraction form was developed *a priori* and piloted on a small sample of included studies before full extraction. Two authors extracted data independently. Extracted datasets were compared for agreement, and discrepancies were resolved by consensus with recourse to a third reviewer when required. When key details were missing or ambiguously reported, attempts were made to contact corresponding authors. If no clarification was obtained, the available data were used and the limitation was documented.

### 2.7 Data items and outcome domains

The following study-level data were extracted: author, year, country, study design, sample size, age, sex or gender distribution, baseline participant characteristics, intervention and comparator details, outcome instruments, assessment time points, adherence or attendance, adverse events, and all results relevant to the review question.

Intervention characteristics included program duration, session frequency, session length, step height where reported, intensity prescription or monitoring method, music use, instructor involvement, group versus home format, and whether the intervention was purely step-aerobic or combined with additional exercise components.

Outcome data were extracted by domain and time point. During synthesis, acute affective states, state anxiety, perceived stress, and mood were interpreted as proximal psychological responses; sleep quality was interpreted as a recovery-related outcome; and self-perceptions, enjoyment, intention, global psychological well-being, and mental-health-related quality-of-life domains were interpreted as broader psychosocial or integrative well-being outcomes. For acute studies, immediate post-session effects were prioritized. For repeated interventions, post-intervention and longest available follow-up results were extracted. Where both total scores and mental-health subscales were reported, the outcome most conceptually aligned with psychological well-being was prioritized.

### 2.8 Risk of bias assessment

Risk of bias in randomized studies was assessed using RoB 2 (Sterne et al., 2019), and risk of bias in nonrandomized intervention studies was assessed using ROBINS-I (Sterne et al., 2016). Assessments were completed independently by two authors at the outcome level where appropriate. Domain-level judgments and overall judgments were reported, and disagreements were resolved by consensus or by third-author adjudication.

### 2.9 Synthesis methods

Results were summarized narratively using a structured Synthesis without meta-analysis-informed approach organized by outcome domain, life-stage population, and intervention time horizon (acute single-session exposure versus repeated intervention). Within each grouping, studies were compared according to design, sample characteristics, intervention features, risk of bias, and direction and precision of findings. Summary tables showed study characteristics, intervention characteristics, outcomes, and key findings in a standardized format.

Because the included studies were heterogeneous in design, comparator type, intervention duration, outcome instruments, and reporting format, a meta-analysis was not considered methodologically appropriate. The synthesis therefore used prespecified narrative decision rules to classify and interpret findings. The primary basis for interpretation was the direction of the between-group effect, or the contrast between step aerobics and the most relevant comparator when a between-group estimate was available. Statistical significance was considered when reported, but it was not used as the sole criterion for interpretation because several studies were small, exploratory, or incompletely reported.

Findings were classified as beneficial when the step-aerobics condition showed a favorable between-group difference or a clearly favorable pre-post change in a study without a comparator. Findings were classified as no clear effect when between-group differences were null, inconsistent, or insufficiently reported to support a directional interpretation. Findings were classified as mixed when favorable and null findings coexisted within the same study, outcome domain, time point, or comparison structure. Findings were classified as adverse or mixed when at least one outcome suggested a less favorable psychological response to step aerobics or to a specific step-aerobics class context. For single-group pre-post studies, favorable within-group change was interpreted cautiously and was not treated as equivalent to controlled evidence.

For studies with multiple outcomes within the same domain, the outcome most directly aligned with the review question and with the study’s stated psychological or psychosocial endpoint was prioritized. Where both total scores and mental-health or psychosocial subscales were reported, the subscale most conceptually aligned with psychological well-being was prioritized over broad physical-health or composite scores. For studies reporting both primary and secondary outcomes, primary psychological outcomes were prioritized when clearly identified by the study authors. If primary outcomes were not identified, validated domain-specific measures were prioritized over exploratory, single-item, or less clearly defined measures.

For acute studies, immediate post-session outcomes were prioritized because they were most aligned with acute psychological response. For repeated interventions, the primary post-intervention assessment was prioritized for the main synthesis. Follow-up assessments were extracted and described when available, but they were interpreted separately from immediate post-intervention effects. When studies reported multiple interim time points, these were used to describe the temporal pattern of response but did not override the post-intervention judgment unless the post-intervention result was unavailable or clearly inconsistent with the repeated-measures pattern.

For studies with multiple comparison groups, the main interpretation prioritized the comparison that best isolated the effect of structured step aerobics from no exercise, usual activity, wait-list, health education, or another inactive or minimally active comparator. When a study compared step aerobics with another active exercise modality, this comparison was interpreted as evidence about relative modality effects rather than as evidence about exercise versus no exercise. When studies compared alternative step-aerobics contexts, such as leadership style, choreography, mirrors, music, or group climate, findings were interpreted as contextual or delivery-moderator evidence rather than as evidence for the overall effectiveness of step aerobics.

Risk of bias was incorporated into interpretation by downgrading confidence in favorable findings from studies at high or serious risk of bias. Accordingly, consistent favorable patterns were described as “signals,” “tendencies,” or “possible benefits” rather than definitive evidence of effectiveness. No formal certainty-of-evidence rating was undertaken because of the small number of studies per domain, heterogeneous designs, and inconsistent reporting of effect estimates.

## 3 Results

### 3.1 Study selection

**Figure 1:**
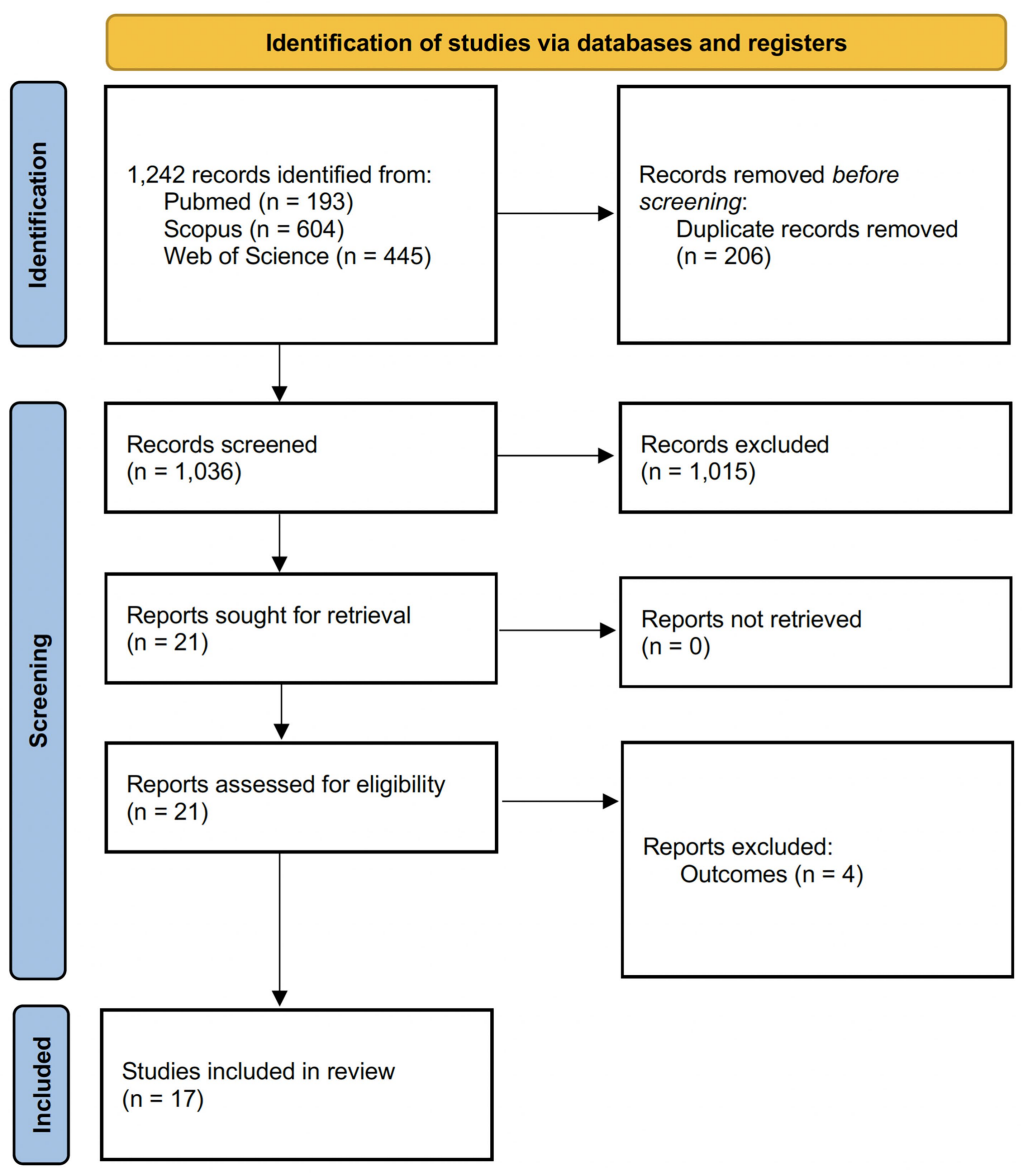
PRISMA flow diagram.

The electronic searches yielded 1,242 imported records across PubMed, Web of Science, and Scopus. After exclusion of duplicate records, 555 unique records were screened by title and abstract. Twenty-one reports proceeded to full-text assessment, and 17 studies were included in the review. Four reports were excluded at the full-text stage because not matching eligibility criteria. Figure 1 presents PRISMA flow diagram.

### 3.2 Study characteristics

Table 2 summarizes the characteristics of the included studies. The evidence base comprised 17 studies published between 1998 and 2023, including 10 randomized studies and 7 nonrandomized studies. Eleven studies evaluated repeated multi-session programs and 6 evaluated acute single-session responses. The populations were concentrated in students and younger adults, midlife or peri-menopausal women, and community-dwelling older adults.

**Table 2:** Characteristics of the included studies.

Study	Population and sample	Design and intervention time horizon	Psychological outcome domain or domains	Classification for synthesis	Assessment time points
Aşçı et al. (1998)	45 sedentary female university students (15 step aerobics; 15 aerobic dance; 15 inactive control)	Randomized parallel-group trial; repeated multi-session	Self-perceptions and body image	Broader psychosocial or quality-of-life-related outcome	Pre-intervention and post 8-week program
Bray et al. (2005)	75 inactive novice female exercisers	Randomized factorial trial; acute single-session	Enjoyment and intention	Broader psychosocial or quality-of-life-related outcome measured immediately after an acute session	Immediate post single 40-min step-aerobics session
Cai et al. (2014)	19 sleep-impaired postmenopausal women (10 step aerobics; 9 matched control)	Nonrandomized controlled study; repeated multi-session	Sleep quality	Recovery-related outcome	Pre-intervention and post-10 weeks (2 days after the program)
Naqvi (2021)	Menopausal women aged 45 to 60 years; step-only arm extracted from a three-arm randomized trial	Randomized parallel-group trial; repeated multi-session	Quality of life and general mental health	Broader psychosocial or quality-of-life-related outcome	Pre-intervention and post-2 weeks
Duman (2013)	48 menopausal women volunteers (24 step aerobics; 24 sedentary control)	Nonrandomized controlled study; repeated multi-session	Depressive symptoms	Broader psychological well-being outcome	Pre-intervention and post-8 weeks
Dunsky et al. (2017)	Community-dwelling older women; step-aerobics arm extracted from a three-arm exploratory trial	Partially randomized controlled study; repeated multi-session	Quality of life and general mental health	Broader psychosocial or quality-of-life-related outcome	Baseline (mean of two pretests), post-8 weeks (48 h after intervention), and 4-week follow-up
Fox et al. (2000)	90 undergraduate students	Randomized factorial trial; acute single-session	Enjoyment and intention	Broader psychosocial or quality-of-life-related outcome measured immediately after an acute session	Immediate post-session
Govindasamy et al. (2023)	60 healthy sedentary obese men aged 18–24 years (20 step aerobics; 20 floor aerobics; 20 inactive control)	Randomized parallel-group trial; repeated multi-session	Psychosocial competence and stress management	Broader psychosocial or quality-of-life-related outcome	Pre-intervention and post 12-week program
Hale and Raglin (2002)	42 college students enrolled in step-aerobics or resistance-training classes	Nonrandomized controlled repeated-measures study; acute single-session	Anxiety and social anxiety	Acute psychological response	Immediately pre-session and within 5 min post-session at weeks 1, 4, and 8 of the course
Hayakawa et al. (2000)	16 middle-aged women already attending bench-stepping classes	Within-participant repeated-measures study; acute single-session	Mood and affect	Acute psychological response	Immediately before and after each of three 60-min bench-stepping sessions held one day apart
Hu et al. (2023)	52 adolescents or young adults with poor sleep (26 step aerobics; 26 control)	Randomized parallel-group trial; repeated multi-session	Sleep quality; Anxiety and social anxiety	Recovery-related outcome; acute or symptom-related psychological response	Baseline, week 4, week 8, and end of week 12 (T + 24 h)
Janyacharoen et al. (2017)	42 healthy older adults	Randomized parallel-group trial; repeated multi-session	Quality of life and general mental health	Broader psychosocial or quality-of-life-related outcome	Baseline and end of 8-week program
Park and Lee (2017)	35 community-dwelling older women	Randomized parallel-group trial; repeated multi-session	Quality of life and general mental health; Self-perceptions and body image	Broader psychosocial or quality-of-life-related outcome	Pre-intervention and post-12 weeks
Lamarche et al. (2009)	51 active female university students	Randomized parallel-group trial; acute single-session	Anxiety and social anxiety; Self-perceptions and body image	Acute psychological response; broader psychosocial or self-evaluative outcome	Immediately pre-session and immediately post-session
Lee and Kim (2007)	28 sedentary overweight or obese adult women with complete post-data (15 step aerobics; 13 control)	Nonrandomized controlled study; repeated multi-session	Self-perceptions and body image; Mood and affect	Broader psychosocial or self-evaluative outcome; psychological response	Pre-intervention and post-12-week program
Martin and Fox (2001)	90 undergraduate students; likely overlapping sample with Fox 2000	Randomized factorial trial; acute single-session	Anxiety and social anxiety	Acute psychological response	Immediate post single 40-min step-aerobics session
Morimura et al. (2015)	36 Japanese returnees from China (30 intervention analyzed; 6 controls)	Nonrandomized controlled study; repeated multi-session	Quality of life and general mental health	Broader psychosocial or quality-of-life-related outcome	Pre-intervention and post-8 weeks

Outcome domains were classified for narrative synthesis according to analytical level: acute psychological responses, recovery-related outcomes, and broader psychosocial or quality-of-life outcomes. Sleep quality was classified as recovery-related rather than as an acute psychological response.

Intervention and comparator details are presented in Table 3. The step-aerobics programs ranged from single classes of approximately 40 min to repeated programs lasting 2–12 weeks. Most repeated interventions were delivered in supervised group formats two or three times per week; several prescribed moderate or moderate-to-vigorous intensity, but reporting of adherence, attendance, instructor behavior, and music characteristics was inconsistent. Comparator conditions were heterogeneous and included inactive controls, health education, other exercise modalities, and alternative step-aerobics contexts such as choreography, mirrors, music, leadership style, or group climate.

**Table 3:** Intervention and comparator characteristics.

Study	Population grouping used in synthesis	Step-aerobics protocol	Comparator context
Aşçı et al. (1998)	Students and younger adults	8 weeks; 3 sessions per week; supervised group classes; 60–70% heart rate reserve	Aerobic dance and inactive control
Bray et al. (2005)	Students and younger adults	Single 40-min step-aerobics class; leadership style and choreography manipulated across eligible step-aerobics conditions	Alternative step-aerobics class conditions
Cai et al. (2014)	Midlife and peri-menopausal women	10 weeks; 3 sessions per week; 40–45 min; group-based; 75–85% heart rate reserve	Matched usual-lifestyle control
Naqvi (2021)	Midlife and peri-menopausal women	2-week step-aerobics program; details incompletely reported	Music therapy alone; step aerobics plus music therapy
Duman (2013)	Midlife and peri-menopausal women	8 weeks; 3 sessions per week; 45–55 min; 40–60% intensity	Sedentary control
Dunsky et al. (2017)	Older adults	8 weeks; group-based program; detailed step prescription incompletely reported	Stability-ball program and ceramics-class control
Fox et al. (2000)	Students and younger adults	Single step-aerobics session; instructor leadership style and group environment experimentally manipulated	Alternative step-aerobics class conditions
Govindasamy et al. (2023)	Students and younger adults	12-week step-aerobics program; reporting indicates supervised training, but adherence and detailed psychological-response scheduling were incompletely described	Floor aerobics and inactive control
Hale and Raglin (2002)	Students and younger adults	Repeated acute assessment during a 50-min class at weeks 1, 4, and 8; target intensity 70 to 80% of individual maximum	Resistance-training class
Hayakawa et al. (2000)	Midlife and peri-menopausal women	Three 60-min bench-stepping sessions under different music conditions on separate days	Alternative music-context step-aerobics conditions
Hu et al. (2023)	Students and younger adults	12 weeks of group step aerobics; sleep assessed at baseline, 4 weeks, 8 weeks, and 12 weeks	Inactive or free-living control
Janyacharoen et al. (2017)	Older adults	8 weeks; 3 sessions per week; 60 min; 18-centimeter one-step platform	General health-education control
Park and Lee (2017)	Older adults	12 weeks; 2 sessions per week; 20–30 min; Korean traditional-dance-based rhythmic step device	Non-exercising control
Lamarche et al. (2009)	Students and younger adults	Single supervised session with 7-min warm-up, 20-min step-aerobics phase, and 7-min cool-down	Mirror-present versus mirror-covered exercise environment
Lee and Kim (2007)	Midlife and peri-menopausal women	12-week bench-step aerobics program	Sedentary overweight or obese control
Martin and Fox (2001)	Students and younger adults	Single 40-min step-aerobics class with manipulated leadership style and group environment	Alternative step-aerobics class conditions
Morimura et al. (2015)	Older adults	8-week program combining home bench-stepping, weekly classes, recreation, pedometer self-monitoring, and instructor feedback	Nonparticipating control group

**Figure 2:**
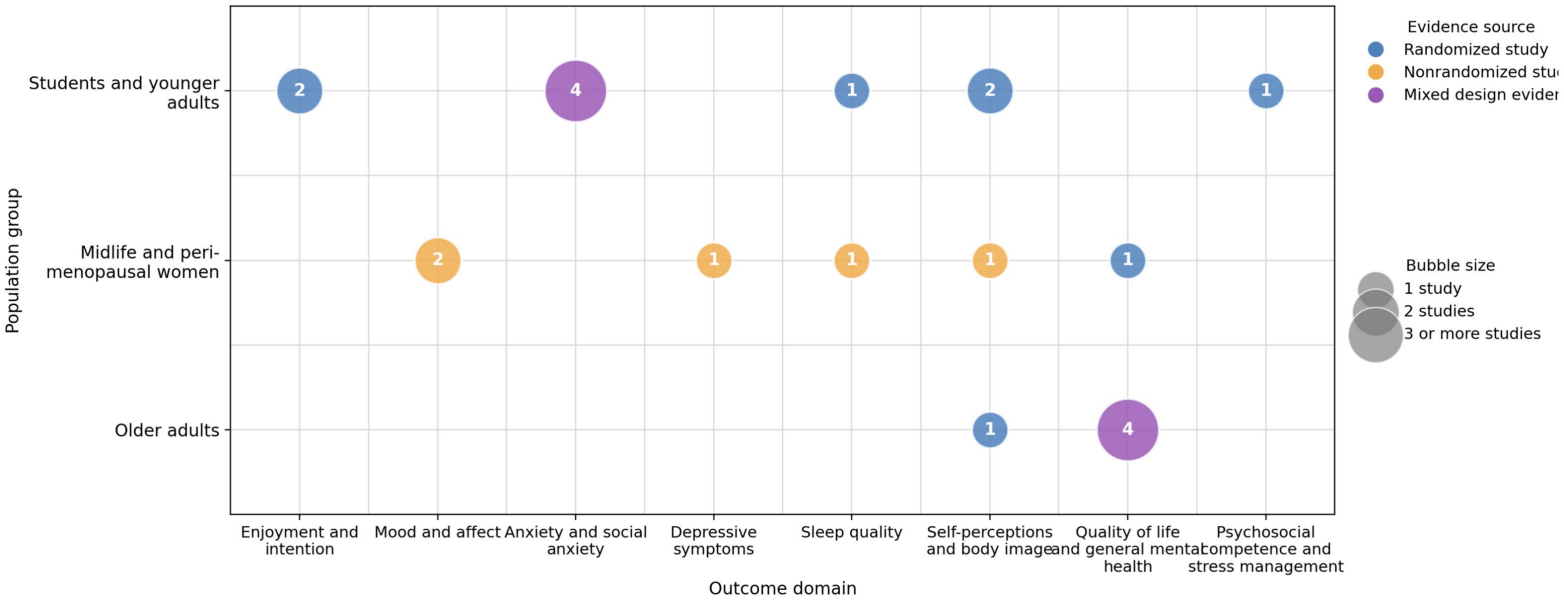
Evidence gap map by population group and outcome domain. Bubble size indicates the number of included studies in each cell, and bubble color indicates whether the evidence in that cell came only from randomized studies, only from nonrandomized studies, or from a mixture of both.

**Figure 3:**
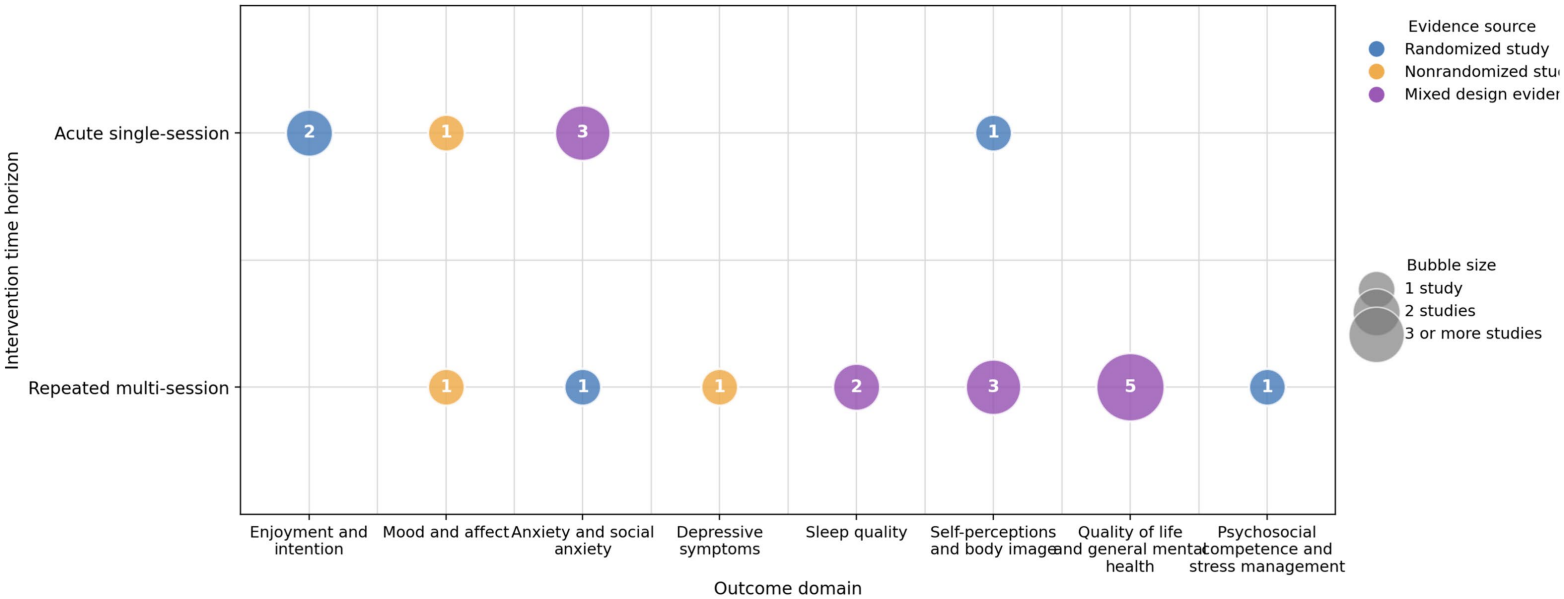
Evidence gap map by intervention time horizon and outcome domain. Bubble size indicates the number of included studies in each cell, and bubble color indicates whether the evidence in that cell came only from randomized studies, only from nonrandomized studies, or from a mixture of both.

Figures 2, 3 translate the included studies into evidence gap maps. The first map shows that the evidence is highly clustered since quality-of-life and general mental-health outcomes were concentrated in older adults, whereas acute anxiety-related and enjoyment studies were concentrated in students and younger adults. The second map shows that repeated multi-session programs dominate the evidence base for sleep, quality of life, body-related outcomes, and depressive symptoms, while acute single-session work is largely limited to enjoyment, mood, and anxiety-related.

### 3.3 Risk of bias in included studies

Risk of bias is presented separately for randomized studies in Table 4 and nonrandomized studies in Table 5. All randomized studies were judged to have high overall risk of bias, most often because subjective self-reported outcomes were measured in unblinded participants and because allocation concealment or prespecified analysis plans were insufficiently described. All nonrandomized studies were judged to have serious overall risk of bias, mainly because of confounding, self-selection into intervention groups, incomplete control of baseline differences, and measurement of subjective outcomes under non-blinded conditions. Accordingly, positive findings were interpreted as hypothesis-generating rather than definitive estimates of effect.

**Table 4:** Risk of bias in randomized studies assessed with risk of bias 2.

Study	Randomization process	Deviations from intended interventions	Missing outcome data	Measurement of outcome	Selection of reported result	Overall judgment
Aşçı et al. (1998)	Some concerns	Some concerns	Low	High	Some concerns	High
Bray et al. (2005)	Some concerns	Low	Low	High	Some concerns	High
Naqvi (2021)	High	Some concerns	Low	High	Some concerns	High
Fox et al. (2000)	Some concerns	Low	Some concerns	High	Some concerns	High
Govindasamy et al. (2023)	Some concerns	Some concerns	Low	High	Some concerns	High
Hu et al. (2023)	Some concerns	Low	Low	High	Some concerns	High
Janyacharoen et al. (2017)	Some concerns	Some concerns	Low	High	Some concerns	High
Park and Lee (2017)	Some concerns	Some concerns	Low	High	Some concerns	High
Lamarche et al. (2009)	Some concerns	Low	Low	High	Some concerns	High
Martin and Fox (2001)	Some concerns	Low	Some concerns	High	Some concerns	High

**Table 5:** Risk of bias in nonrandomized studies assessed with risk of bias in non-randomized studies of interventions.

Study	Confounding	Selection of participants	Classification of interventions	Deviations from intended interventions	Missing data	Measurement of outcomes	Selection of reported result	Overall judgment
Cai et al. (2014)	Serious	Serious	Low	Low	Low	Serious	Moderate	Serious
Duman (2013)	Serious	Serious	Low	Moderate	Low	Serious	Moderate	Serious
Dunsky et al. (2017)	Serious	Serious	Low	Moderate	Moderate	Serious	Moderate	Serious
Hale and Raglin (2002)	Serious	Serious	Low	Moderate	Serious	Serious	Moderate	Serious
Hayakawa et al. (2000)	Serious	Moderate	Low	Moderate	Low	Serious	Moderate	Serious
Lee and Kim (2007)	Serious	Serious	Low	Moderate	Serious	Serious	Moderate	Serious
Morimura et al. (2015)	Serious	Serious	Low	Moderate	Serious	Serious	Moderate	Serious

### 3.4 Results of individual studies and narrative synthesis

Table 6 summarizes the main findings of the individual studies. Across the full set of included studies, the most frequently reported favorable findings concerned sleep quality, depressive symptoms in one menopause-focused study, selected quality-of-life or general mental-health outcomes, and some acute anxiety-related responses. However, these findings were derived from small, heterogeneous studies with elevated risk of bias and should be interpreted as preliminary. By contrast, self-perceptions, body image, enjoyment, and socially evaluative responses were more context dependent, and evidence for psychosocial competence or stress-management outcomes was sparse and inconclusive.

**Table 6:** Summary of the main findings of the individual studies.

Study	Outcome domain or domains	Main review-relevant findings	Interpretation
Aşçı et al. (1998)	Self-perceptions and body image	No statistically significant adjusted between-group differences were reported for physical self-perception or body image satisfaction	No clear effect
Bray et al. (2005)	Enjoyment and intention	More supportive leadership and more varied choreography improved post-session enjoyment; intention to attend future classes did not differ clearly across conditions	Mixed
Cai et al. (2014)	Sleep quality	Sleep quality improved after the training program relative to matched control; the report also described increased melatonin levels	Beneficial
Naqvi (2021)	Quality of life and general mental health	The step-only arm improved on the total menopause-specific quality-of-life score from 32.42 to 28.74, but comparative advantage over the alternative arms was not established and music therapy alone appeared more favorable	Mixed
Duman (2013)	Depressive symptoms	Depressive symptom scores decreased after the program, alongside favorable body-composition change	Beneficial
Dunsky et al. (2017)	Quality of life and general mental health	Improvements were reported in general health perception and role limitation due to emotional state, with broader quality-of-life effects not uniformly consistent across domains or follow-up	Mixed
Fox et al. (2000)	Enjoyment and intention	Enjoyment was higher when supportive leadership was combined with a more supportive group environment, and probability of future involvement was higher in the enriched group environment	Beneficial
Govindasamy et al. (2023)	Psychosocial competence and stress management	The report did not show clear statistically significant advantages for self-confidence, emotional adjustment, assertiveness, interpersonal relationships, or stress management	No clear effect
Hale and Raglin (2002)	Anxiety and social anxiety	State anxiety decreased from pre-class to post-class across the training period after step-aerobics sessions, consistent with recurrent acute anxiolytic effects	Beneficial
Hayakawa et al. (2000)	Mood and affect	Bench-stepping performed with music was associated with more favorable mood responses than the no-music condition, including less fatigue and confusion and more vigor in some comparisons	Beneficial
Hu et al. (2023)	Sleep quality; Anxiety and social anxiety	Sleep quality improved from week 8 onward and remained better by week 12; anxiety was measured, but the comparative trial reporting emphasized sleep outcomes more strongly than anxiety results	Beneficial
Janyacharoen et al. (2017)	Quality of life and general mental health	Quality-of-life scores were higher after the stepping program than in the control group at study end	Beneficial
Park and Lee (2017)	Quality of life and general mental health; Self-perceptions and body image	Health-related quality of life and exercise self-efficacy both improved after the program	Beneficial
Lamarche et al. (2009)	Anxiety and social anxiety; Self-perceptions and body image	Self-presentational efficacy increased and state social anxiety decreased from pre-session to post-session, but no clear between-group effect of mirrors was observed	Mixed
Lee and Kim (2007)	Self-perceptions and body image; Mood and affect	Body image improved relative to control, whereas anger, exercise self-efficacy, and exercise-related affect did not show clear between-group differences	Mixed
Martin and Fox (2001)	Anxiety and social anxiety	Participants in the enriched group condition reported higher social anxiety than those in the bland group condition, indicating that some socially salient class climates may intensify rather than relieve social-evaluative concerns	Adverse or mixed
Morimura et al. (2015)	Quality of life and general mental health	General mental health scores and the mental component of quality of life improved after the program; the matched-control comparison supported improvement in the mental component score and suggested improvement in general mental health	Beneficial

“Beneficial” indicates a favorable between-group difference or, in uncontrolled studies, a favorable pre-post change interpreted cautiously. “No clear effect” indicates null, inconsistent, or insufficiently reported findings. “Mixed” indicates coexistence of favorable and null findings within the same study, outcome domain, time point, or comparison structure. “Adverse or mixed” indicates that at least one outcome suggested a less favorable psychological response to step aerobics or to a specific step-aerobics class context.

## 4 Discussion

The main finding from the current systematic review is that the evidence base is small, methodologically heterogeneous, and at consistently elevated risk of bias, which prevents firm conclusions about the effectiveness of structured step aerobics for psychological well-being. Because all randomized studies were judged to have high overall risk of bias and all nonrandomized studies were judged to have serious overall risk of bias, the findings across all outcome domains should be interpreted primarily as hypothesis-generating rather than as evidence of intervention effectiveness. Within this uncertain evidence base, favorable findings were most often reported for sleep quality, selected broad quality-of-life or general mental-health outcomes, depressive symptoms in a single menopause-focused study, and some immediate anxiety-related responses after step-aerobics sessions. However, these findings may reflect bias from inadequate allocation concealment, lack of blinding for subjective outcomes, confounding, self-selection, incomplete reporting, or selective outcome emphasis, rather than true intervention effects. By contrast, outcomes such as body-related self-perceptions, self-efficacy, enjoyment, and socially evaluative responses were more contingent on class context, comparator choice, and population, with positive and null findings coexisting across studies.

### 4.1 Acute responses and class-context factors

The acute evidence provides only limited and preliminary support for immediate psychological responses to step aerobics, and the direction of these responses appears strongly context dependent. Enjoyment outcomes were reported as more favorable in some studies when the session was delivered with supportive leadership, richer group processes, or more engaging choreography, suggesting cautiously that the psychosocial climate of the class may be part of the intervention context rather than a peripheral feature (Fox et al., 2000; Bray et al., 2005). A study (Hayakawa et al., 2000) similarly showed that music context can shape acute mood responses during bench stepping, reinforcing the idea that step aerobics is a composite experience involving rhythm, cueing, social atmosphere, and movement rather than a purely metabolic dose of exercise. These findings fit with broader work on group-based exercise, which has emphasized enjoyment, social connectedness, and class climate as important pathways through which exercise becomes psychologically meaningful and behaviorally sustainable (Beauchamp et al., 2018; Gwyther et al., 2018).

At the same time, the acute anxiety-related evidence shows that psychologically supportive class features are not invariably beneficial for every participant or every construct. A study (Hale and Raglin, 2002) reported repeated immediate reductions in state anxiety after step-aerobics sessions across an eight-week class period, and other (Lamarche et al., 2009) suggested that a single class can improve self-presentational efficacy without producing adverse socially evaluative reactions overall. However, a study (Martin and Fox, 2001) found higher state social anxiety in a more socially salient class environment, and others (Fox et al., 2000; Bray et al., 2005) showed that greater enjoyment did not necessarily translate into a comparably strong increase in intentions for future participation. Given the high or serious risk of bias, small samples, and reliance on subjective outcomes measured immediately after exposure, these studies should be interpreted as showing that acute step-aerobics sessions may be experienced favorably in some contexts, rather than as demonstrating reliable acute psychological benefit. The same features that make classes engaging may also heighten self-awareness or social comparison in some contexts. The implication is that acute psychological benefit is not determined by movement content alone and it is possibly shaped by how visible, performative, and socially evaluative the class becomes.

This acute literature nevertheless remains limited in what it can tell us about meaningful mental-health change. Most acute studies were conducted in student or young adult samples, relied on single-session designs, and used subjective outcomes measured immediately after exposure (Fox et al., 2000; Martin and Fox, 2001; Hale and Raglin, 2002; Bray et al., 2005; Lamarche et al., 2009). Those designs are useful for understanding acceptability, immediate affect, and the likely experiential ingredients of a successful session, but they are a weak basis for inferring durable improvements in psychological well-being. The immediate evidence is therefore best interpreted as preliminary acceptability and experiential evidence, suggesting that some participants may report favorable in-session responses, while leaving open whether these responses are reproducible, attributable to step aerobics itself, or capable of accumulating into sustained psychological benefits over time.

### 4.2 Repeated interventions: sleep, quality of life, and general mental health

The most frequently reported favorable findings in the repeated-intervention literature concerned sleep and broader perceived well-being, but the certainty of this pattern is low. Two studies (Cai et al., 2014; Hu et al., 2023) reported improved sleep quality after multi-week step-aerobics programs in participants with poor baseline sleep, despite differences in age and setting. The convergence of these two studies is worth noting because sleep was one of the few domains in which the reported direction of change was aligned across more than one investigation, although both studies had important methodological limitations. Even so, the sleep literature remains too small to support firm effectiveness claims. Both studies used self-reported sleep outcomes, both focused on selected subgroups with sleep difficulties rather than general community samples, and neither study resolved the broader methodological concerns that affected the rest of the evidence base. A more cautious interpretation is that sleep quality is one of the few domains in which preliminary favorable findings have been reported in more than one study, but the high or serious risk of bias, self-reported sleep measures, selected poor-sleep samples, and limited replication prevent any conclusion that step aerobics is sleep-supportive.

Broad quality-of-life and general mental-health outcomes also showed some favorable findings in older adult populations, but these findings are difficult to separate from nonspecific effects of supervised group participation, expectancy, social contact, baseline imbalance, self-selection, and subjective outcome measurement. Therefore, they should not be interpreted as reliable evidence that step aerobics improves quality of life or general mental health. Two studies (Janyacharoen et al., 2017; Park and Lee, 2017) reported improvements in broad quality-of-life measures, while other (Dunsky et al., 2017) described benefits in some domains such as general health perception and emotional role limitation rather than across the full profile of psychosocial outcomes. A study (Morimura et al., 2015) extended this pattern by reporting better general mental health and a more favorable mental-component quality-of-life score after a bench-stepping support program. Across these studies, the signal is relevant because it points less to narrow symptom reduction than to broader perceived functioning and well-being. That pattern is also broadly compatible with the wider exercise literature, in which psychological benefits are often expressed through mood, vitality, and mental-health-related quality of life rather than through large changes in a single diagnostic symptom scale (Penedo and Dahn, 2005; Koch et al., 2019; Mahindru et al., 2023; Singh et al., 2023).

By contrast, evidence for more specific psychosocial and self-perceptual outcomes was inconsistent. A study (Duman, 2013) reported lower depressive symptoms after an eight-week program in menopausal women, but this domain was not replicated by another directly comparable study. A study (Aşçı et al., 1998) found no clear benefit for physical self-perception or body-image satisfaction, whereas other (Lee and Kim, 2007) observed a more favorable body-image pattern but not corresponding advantages for anger, exercise-related affect, or exercise self-efficacy. A study (Park and Lee, 2017) reported improved exercise self-efficacy in older women, yet other (Govindasamy et al., 2023) found no clear advantages for self-confidence, emotional adjustment, assertiveness, interpersonal relationships, or stress management. A study (Naqvi, 2021) reported improvement in a broad menopause-specific quality-of-life score in the step-aerobics-only arm, but the construct was multidimensional and the comparative pattern did not isolate step aerobics as the clearly superior component. Thus, the limited repeated-intervention evidence contains more frequently reported favorable findings for sleep and broad perceived well-being than for finely grained constructs such as body image, psychosocial competence, or stress-management capacity, but the risk-of-bias profile prevents these patterns from being interpreted as comparative evidence of stronger effects in these domains.

### 4.3 Population distribution and intervention-delivery features

An important contribution of this review is that it clarifies where the evidence is concentrated and where it is sparse. Acute studies were clustered in students and younger adults, whereas repeated programs contributing quality-of-life and mental-health outcomes were concentrated in older women and other midlife or older-adult samples. Sleep outcomes were studied only in selected poor-sleep groups, and there was very little evidence in men, mixed community adult samples, or younger adolescents outside the sleep literature. This uneven distribution matters because it limits the external validity of the field. The preliminary favorable findings may partly reflect the fit between the intervention and the population studied, such as older adults participating in socially structured group exercise or women engaging in programs that also support routine, mastery, and perceived vitality. What remains unclear is whether comparable effects would be seen in broader community populations, in men, or in groups with more diverse baseline mental-health profiles.

The review raises a hypothesis, based on limited and bias-prone evidence, that intervention design may influence outcomes in at least two ways. First, repeated programs showing favorable findings were commonly delivered over 8–12 weeks and usually in supervised group formats, which may allow benefits to accrue through repeated exposure, social reinforcement, and behavioral regularity rather than through any single session alone (Duman, 2013; Cai et al., 2014; Morimura et al., 2015; Janyacharoen et al., 2017; Park and Lee, 2017; Hu et al., 2023). Second, the acute studies indicate that music, choreography, instructor style, mirrors, and group climate can materially alter how the same underlying exercise dose is experienced (Fox et al., 2000; Hayakawa et al., 2000; Martin and Fox, 2001; Bray et al., 2005; Lamarche et al., 2009). That observation is consistent with the conceptual rationale for treating step aerobics as a distinct exercise modality rather than as generic aerobic activity. However, reporting of adherence, attendance, step height, progression, instructor behavior, and music characteristics was often incomplete, making it impossible to identify a robust set of active ingredients. The current literature therefore identifies program design as a plausible moderator requiring direct testing, but it does not establish which delivery features are active, necessary, or sufficient for psychological benefit.

### 4.4 Risk of bias, evidence limitations, and research priorities

The main limitation of the evidence base is the consistently elevated risk of bias. Every randomized study was judged to have high overall risk of bias, and every nonrandomized study was judged to have serious overall risk of bias. In randomized studies, the most common problems were insufficient reporting of allocation concealment or analysis planning and the use of subjective self-reported outcomes in participants who were fully aware of their intervention condition. In nonrandomized studies, confounding and self-selection were the dominant concerns, because participants often entered exercise programs by preference or were compared with separately recruited controls. Across designs, the literature was further weakened by small samples, limited adherence reporting, inconsistent handling of missing data, short follow-up, and reliance on broad composite outcomes that did not always isolate the psychological domain of greatest interest. These weaknesses mean that favorable results should be interpreted as signals of possible benefit rather than trustworthy estimates of effect size. In addition, the included outcomes operated at different analytical levels, ranging from immediate affective or anxiety responses to recovery-related sleep outcomes and broader psychosocial or quality-of-life domains. This prevented any defensible pooled interpretation of psychological well-being as a single homogeneous endpoint.

The review itself also has limitations that should be acknowledged. The field was too heterogeneous in design, population, comparator structure, and outcome measurement to support a meaningful meta-analysis, so the conclusions necessarily rely on structured narrative comparison. Although explicit synthesis decision rules were applied, the small number of studies per domain and incomplete reporting of effect estimates meant that several judgments necessarily relied on qualitative interpretation rather than formal quantitative pooling. Some potentially relevant reports were unavailable as complete full texts and therefore could not be fully extracted or appraised, which may have reduced coverage of older step-aerobics literature. In addition, several studies reported psychological outcomes only as part of wider health or fitness papers, and effect estimates were not always recoverable in sufficient detail for stronger comparative inference. These constraints do not invalidate the synthesis, but they do reinforce that the present discussion should be read as a careful mapping and interpretation of an incomplete literature rather than as a definitive effectiveness statement.

Future research should respond directly to these design weaknesses. The field needs adequately powered randomized trials with transparent sequence generation and allocation concealment, prospectively specified analyses, and longer follow-up beyond the end of the program. Outcomes should be selected from a clearer core set of psychological domains, with validated instruments and reporting that separates broad quality-of-life composites from more specific mental-health constructs. Trials should also report adherence, attendance, progression, adverse events, and key protocol features such as step height, intensity prescription, choreography complexity, use of music, and instructor training. Where possible, future studies should compare step aerobics not only with inactive controls but also with other exercise modalities and with alternative step-aerobics delivery styles, so that the field can begin to distinguish exercise dose effects from modality-specific contextual effects. Finally, the population base should widen to include men, mixed community adult cohorts, younger participants outside selected sleep samples, and culturally diverse settings.

### 4.5 Provisional implications and future directions

Given the methodological limitations, the practical implications of this review should be considered provisional. Structured step aerobics should not yet be recommended as an evidence-based intervention for improving psychological well-being, but it remains a feasible community exercise format worthy of more solid evaluation, particularly for outcomes such as sleep quality, broader perceived well-being, and acute exercise experience. The available evidence also raises the possibility that delivery context matters. Future interventions may be more acceptable if instructors create a socially supportive environment, progression is manageable, and the session design promotes enjoyment without making the environment unnecessarily evaluative or intimidating (Fox et al., 2000; Martin and Fox, 2001; Bray et al., 2005). For older adults and novice participants in particular, future trials should evaluate step aerobics within structured, inclusive, and well-supervised programs rather than assuming that performance-oriented class formats will produce comparable psychological responses.

The next practical step for the research agenda is to use the evidence gap structure identified in this review to prioritize tractable questions rather than to continue producing small, weakly reported studies across unrelated outcomes. Sleep, broad quality of life, and acute affective experience are the domains with the clearest initial signal and therefore the best candidates for replication. Equally important, null or mixed domains such as body image, self-efficacy, and stress-management outcomes should not be abandoned; they should be tested with stronger designs and more precise constructs. Evidence gap mapping is particularly useful here because it shows that the problem is not simply whether step aerobics works, but for whom, under what class conditions, over what time horizon, and for which psychological outcomes. A more strategically cumulative program of research would make the next review substantially more decisive than the present one.

## 5 Conclusion

This review found insufficient evidence to determine whether structured step aerobics improves psychological well-being in nonclinical populations. The limited evidence is most hypothesis-generating for sleep quality, broader perceived well-being or general mental health, and acute psychological responses, while acute studies raise the possibility that enjoyment, anxiety-related responses, and other immediate experiences may be shaped by the social and instructional features of the class. However, the small number of studies, the reliance on subjective outcomes, the short follow-up periods, and the uniformly elevated risk of bias mean that no firm claim can yet be made about effectiveness, superiority over other exercise modalities, or the optimal way to design a step-aerobics program for psychological benefit. The principal contribution of the review is therefore not to establish effectiveness, but to provide a modality-specific map of the step-aerobics evidence base, identify where psychological and psychosocial evidence is concentrated, clarify where gaps remain, and indicate which next studies would be most valuable for moving the field toward credible inference.

## Data Availability

The original contributions presented in the study are included in the article/supplementary material, further inquiries can be directed to the corresponding author.
